# Effects of Supplementation with Dimethylglycine Sodium Salt on Immunity, Intestinal Tissue Morphology, and Antioxidant Function in IUGR Lambs

**DOI:** 10.3390/ani16081258

**Published:** 2026-04-20

**Authors:** Yuwei Wang, Mengfei Li, Lin Ma, Yurong Lin, Cheng Zhang, Zhiqiang Cheng, Yong Chen, Changjiang Zang

**Affiliations:** Xinjiang Herbivore Nutrition Laboratory for Meat & Milk, College of Animal Science, Xinjiang Agricultural University, Urumqi 830052, China; wywwyw3000@126.com (Y.W.); lmf0212@126.com (M.L.); 15228902461@163.com (L.M.); 19193552979@163.com (Y.L.); zc9624641123@163.com (C.Z.); cheng07162022@163.com (Z.C.); xjaucy@163.com (Y.C.)

**Keywords:** dimethylglycine sodium salt, IUGR, growth performance, intestinal tissue morphology, antioxidant capacity

## Abstract

This study investigates the regulatory effects of dietary supplementation with DMG-Na on growth performance, immunity, and intestinal development in IUGR lambs. A total of 45 lambs were used: thirty IUGR (3.10 ± 0.16 kg) lambs were randomly assigned to IUGR or IUGR + DMG-Na (0.1% in milk replacer from days 7–56) groups, with fifteen normal birth weight lambs as CON (4.32 ± 0.17 kg). At 56 days of age, eight lambs per group were slaughtered for sample collection. Compared to CON, IUGR lambs showed a significantly lower final body weight and average daily gain (ADG) (*p* < 0.01); IUGR also severely compromised intestinal structure, markedly decreasing villus height and villus height-to-crypt depth ratio across all small intestinal segments (*p* < 0.01); immune function was impaired, with highly significantly lower jejunal secretory IgA (sIgA) (*p* < 0.01); and antioxidant capacity was diminished, evidenced by reduced jejunal GSH, catalase (CAT) and glutathione reductase (GR) activities (*p* < 0.05) and increased jejunal MDA content (*p* < 0.01). Compared to IUGR, IUGR + DMG-Na group had highly significant increased final body weight and significant increased ADG (*p* < 0.01); it enhanced intestinal morphology, notably increasing villus height and villus height-to-crypt depth ratio in the duodenum and jejunum (*p* < 0.01); immune markers improved, with elevated jejunal sIgA (*p* < 0.05); and antioxidant status was restored, demonstrated by increased jejunal GSH and CAT activities (*p* < 0.05) and decreased jejunal MDA content (*p* < 0.01). In conclusion, DMG-Na effectively counteracted IUGR-induced deficits by promoting intestinal development, immunity, and antioxidant capacity, ultimately improving growth performance.

## Simple Summary

Intrauterine growth restriction (IUGR) refers to the impaired growth and development of fetuses during pregnancy, resulting in low birth weight and long-lasting negative effects on health and performance in offspring. This study examined whether dietary supplementation with dimethylglycine sodium salt (DMG-Na) could enhance growth, immunity, intestinal health, and antioxidant capacity in IUGR lambs. Feeding IUGR lambs milk replacer containing 0.1% DMG-Na alleviated these deficits: it promoted growth and organ development (liver and spleen), restored intestinal structure and digestive enzyme activity, and strengthened both immune function and antioxidant capacity. These findings suggest that DMG-Na supplementation is an effective nutritional strategy to mitigate the adverse effects of IUGR in lambs, potentially improving their health and productivity in livestock production.

## 1. Introduction

IUGR refers to impaired fetal growth, characterized by slower growth rates and failure to achieve the expected weight for gestational age [1,2,3]. IUGR increases the risk of preterm birth and stillbirth, leading to reduced neonatal survival rates and permanent postnatal growth retardation. Moreover, it compromises nutritional efficiency, elevating the risk of cardiovascular and metabolic diseases in adulthood [3,4].

In addition, IUGR diminishes antioxidant capacity, making animals more susceptible to oxidative stress [5]. Abnormalities in the intestinal redox state may compromise the health of IUGR neonates in the early postnatal period and may be associated with adult disease risk [6,7]. Oxidative damage arises from dysregulation of antioxidant and free radical generation systems, which elevates reactive oxygen species (ROS) levels, ultimately causing tissue and mitochondrial injury [8]. The structure and function of ruminant intestinal tissues are critical for nutrient digestion and absorption [9]. The small intestine is vital for nutrient digestion, absorption, and metabolism, and also serves as the primary immune organ in animals [10]. Intestinal morphology is a key indicator of gut health and function [11]. IUGR significantly impacts neonatal intestinal development and barrier function, leading to long term growth impairment typically manifested as reduced villus height and width [12]. These structural alterations compromise small intestinal function, impairing nutrient absorption and consequently affecting overall health status.

Dimethylglycine (N,N-dimethylglycine, DMG) is a naturally occurring non protein amino acid that serves as an intermediate in the metabolic conversion of choline to glycine [13,14]. In animals, betaine is a metabolite of choline; under the action of betaine homocysteine methyltransferase, betaine transfers a methyl group to homocysteine, producing methionine and DMG [15]. As a methyl donor, DMG participates in the methionine cycle, contributing to the synthesis of S-adenosylmethionine (SAM). SAM is the primary methyl donor for DNA, RNA, protein, and lipid methylation, which are essential for regulating gene expression, cell proliferation, and the maintenance of intestinal epithelial barrier function—all of which are impaired in IUGR [14,15]. Importantly, the methyl donor function of DMG is intimately linked to glutathione (GSH) synthesis through the transsulfuration pathway [16]. After donating its methyl group, SAM is converted to S-adenosylhomocysteine (SAH) and then to homocysteine [17]. Instead of being remethylated to methionine, homocysteine can enter the transsulfuration pathway, where it is irreversibly converted to cysteine via cystathionine β-synthase (CBS) and cystathionine γ-lyase (CTH) [18]. Cysteine is the rate-limiting substrate for GSH synthesis. Therefore, by fueling the methionine cycle and subsequently the transsulfuration pathway, DMG indirectly increases cysteine availability, thereby promoting GSH production [15]. Additionally, DMG itself is metabolized to glycine, another direct precursor of GSH. Thus, DMG supports GSH synthesis through two complementary mechanisms: (1) as a methyl donor that drives the transsulfuration pathway to generate cysteine; (2) as a direct source of glycine. DMG is a lipophilic, water-soluble small molecule that readily crosses cell membranes and is rapidly and completely absorbed after oral administration [15]. Dimethylglycine sodium salt (DMG-Na) is the sodium salt form of DMG, that enhances molecular stability and physicochemical properties [19]. DMG-Na is absorbed in the gut, transported to the liver via the portal system, and distributed throughout tissues and organs via the bloodstream, where it exerts antioxidant and methyl-donor effects [15]. We hypothesized that dietary supplementation with DMG-Na would improve growth performance and intestinal development in IUGR lambs, and that these beneficial effects are mediated by DMG-Na’s role as a glutathione precursor and antioxidant that maintains intestinal morphology and protects against oxidative damage.

## 2. Materials and Methods

### 2.1. Ethical Considerations

All animal care and handling procedures adhered to the Guidance of the Care and Use of Laboratory Animals in China and were approved by the Animal Care Committee of Xinjiang Agricultural University (Animal protocol number: 2023055).

### 2.2. Experimental Materials

Dimethylglycine sodium salt (C_4_H_8_NNaO_2_, DMG-Na) used in this trial, with a purity of 95.0%, was purchased from Shaanxi Dideu Medichem Co., Ltd., Xi’an, China. Milk replacer was purchased from Beijing Precision Animal Nutrition Research Center, Beijing, China.

### 2.3. Experimental Design

Hu sheep ewes that had given birth to triplets (obtained from Xinjiang Shangpin Meiyang Technology Co., Ltd., changji, China) were selected based on parity, body weight, and age at lambing. From their offspring, a total of 45 male Hu sheep lambs were selected for the 49-day trial period. IUGR can be defined as fetal or birth weight less than 2 standard deviations (SD) of the mean body weight for gestational age [19]. A total of 30 IUGR lambs (birth weight 3.10 ± 0.16 kg) were included in the study. To ensure balanced allocation, the lambs were first sorted from lowest to highest birth weight. They were then evenly allocated to the IUGR group (milk replacer without DMG-Na, *n* = 15) and the IUGR + DMG-Na group (milk replacer supplemented with 0.1% DMG-Na, *n* = 15) using a random number table. The allocation process was performed independently by a researcher who was not involved in subsequent sample collection, data acquisition, and laboratory analyses. An additional fifteen male Hu sheep lambs with an average birth weight of 4.32 ± 0.17 kg were selected as the normal birth weight control group (CON, *n* = 15) and received supplementary milk replacer without DMG-Na.

### 2.4. Experimental Animal Treatments

The experimental lambs were housed with their dams in the same elevated pens with slatted floors, with ad libitum access to water. Pens were cleaned at regular intervals. Routine vaccinations and periodic disinfection were carried out according to the farm’s standard husbandry protocols throughout the trial period. Lambs were allowed to suckle freely with their ewes from 0 to 7 days of age. From 8 to 56 days of age, lambs were supplemented with milk replacer at 2% of the average body weight of all lambs per day, divided into two equal feedings (09:00 and 16:00), while having ad libitum access to starter feed throughout the experimental period [20]. Starter feed supplementation commenced at 8 days of age. Lambs were weighed weekly, and the feeding amount was adjusted accordingly based on the most recent body weight. Prior to feeding, the milk replacer (with or without 0.1% DMG-Na) was thoroughly mixed with water at 50 °C at a ratio of 1:6 to achieve a dry matter (DM) content of 16.67% [21], which approximates the dry matter content found in ewe milk. The mixture was maintained at 40 °C in a water bath and then transferred into feeding bottles before administration. The supplementation level of DMG-Na was determined based on previous studies in piglets [8,22]. The nutritional composition of both the milk replacer and starter feed met the requirements recommended by the NRC Nutrition Guidelines [23]. The nutritional content levels of lamb starter feed are listed in Table 1.

### 2.5. Sample Collection

On day 49 of the trial, the lambs were fasted for 2 h before slaughter (from 9:00 a.m. to 11:00 a.m.) and water was provided ad libitum. Blood samples were collected before the morning feeding. A total of 5 mL of blood was collected from each lamb into sterile tubes and allowed to clot at room temperature. Serum was separated by centrifugation at 3500 r/min for 15 min at 4 °C. The supernatant was transferred into 1.8 mL Eppendorf tubes and stored at −80 °C until analysis of serum antioxidant enzyme activities and other biochemical parameters. After blood collection, eight lambs per group were deeply anesthetized with an intravenous injection of sodium pentobarbital (100 mg/kg body weight) and slaughtered. The abdominal cavity was immediately opened, and the heart, liver, spleen, lungs, and kidneys were rapidly excised and were weighed. Tissue samples from the duodenum, jejunum, and ileum were collected, flushed with ice-cold physiological saline to remove digesta, and fixed in 4% paraformaldehyde solution for subsequent paraffin sectioning. For jejunal mucosa collection, a segment of the jejunum was opened longitudinally, rinsed with ice-cold physiological saline to remove digesta, and laid flat on an ice-cold plate. The mucosal layer was gently scraped off using a sterile glass slide, transferred into 2.0 mL RNase-free tubes, and immediately snap-frozen in liquid nitrogen before storage at −80 °C for subsequent analysis of antioxidant enzyme activities and related parameters.

### 2.6. Sample Determination and Analysis

#### 2.6.1. Growth Performance

Lambs were weighed every 7 days. Initial and final body weights were evaluated for growth performance. Growth performance is calculated using the following formula:

$$\mathrm{Average}\;\mathrm{Daily}\;\mathrm{Gain}\;(\mathrm{ADG},\;g/d)\;=\;\frac{\mathrm{Final}\;\mathrm{weight}\;\left(\mathrm{kg}\right)-\mathrm{Initial}\;\mathrm{weight}\;(\mathrm{kg})}{\mathrm{Number}\;\mathrm{of}\;\mathrm{trial}\;\mathrm{days}\;(d)}\;\times \;1000$$

#### 2.6.2. Organ Indices

After slaughter, the heart, liver, spleen, lungs, and kidneys were excised and weighed individually. Organ indices were calculated as follows:

$$\mathrm{Organ}\;\mathrm{Index}\;(\%)\;=\;\frac{\mathrm{Organ}\;\mathrm{weight}\;(g)}{\mathrm{Preslaughter}\;\mathrm{live}\;\mathrm{weight}}\;\times \;100$$

#### 2.6.3. Histomorphological Observation

Tissue samples from the duodenum, jejunum, and ileum previously fixed in 4% paraformaldehyde solution were rinsed under running tap water for 8 h. Following rinsing, the samples were dehydrated through a graded series of ethanol and xylene, and then embedded in paraffin at 53–56 °C. After embedding, the paraffin blocks were stored overnight at 4 °C. The next day, sections (5 μm thick) were cut using a microtome, mounted on glass slides, dried, and stained with hematoxylin and eosin (H&E). The intestinal tissue sections were digitally scanned using a Motic EasyScanner (Motic China Group Co., Ltd., Xiamen, China), processed using the Motic DSAssistant (x86) and morphometric measurements were performed using ImageJ software version Fiji (National Institutes of Health, Bethesda, MD, USA).

The following histomorphological parameters were measured: small intestinal (duodenum, jejunum, ileum) villus height, villus width, crypt depth, and villus height to crypt depth ratio.

Villus height-to-crypt depth ratio = Villus height/Crypt depth

#### 2.6.4. Immunoglobulin Assay, Endotoxin, and IGF-II

Serum concentrations of immunoglobulin A (IgA), immunoglobulin M (IgM), and immunoglobulin G (IgG) were determined using commercial kits (Nanjing Jiancheng Bioengineering Institute, Nanjing, China) according to the manufacturer’s instructions.

For jejunal mucosa, tissue samples were accurately weighed and homogenized in ice-cold physiological saline at a ratio of 1:10 (*w*/*v*) using a high-speed homogenizer at 2500 rpm for 10 min. The homogenate was then centrifuged at 3500 r/min for 10 min at 4 °C, and the supernatant was collected. The concentration of secretory IgA (sIgA) in the jejunal mucosal homogenate was measured using a commercial kit (Nanjing Jiancheng Bioengineering Institute, Nanjing, China) following the manufacturer’s protocol.

Serum samples (0.05 mL) were diluted 10-fold and heated at 70 °C for 10 min, then cooled. Endotoxins were measured using a chromogenic LAL assay. Controls, standards, and samples were mixed with LAL reagent (100 µL) and incubated at 37 °C for 11 min. Chromogenic substrate (100 µL) was added and incubated for another 6 min. Then, 500 µL each of three diazotization reagents were added. After 5 min, OD was read at 545 nm. Endotoxin concentration was calculated from a standard curve and multiplied by the dilution factor.

The content of IGF-II in serum was determined using a commercial ELISA kit (Nanjing Jiancheng Bioengineering Institute) according to the manufacturer’s protocol.

#### 2.6.5. Determination of Jejunal Digestive Enzyme Activity

Jejunal amylase activity was determined using the iodine-starch colorimetric method. The activities of lipase, maltase, sucrase, trypsin, and lactase were measured using enzyme-linked immunosorbent assay (ELISA) kits. All analyses were performed by the Nanjing Jiancheng Bioengineering Institute according to the manufacturer’s instructions.

#### 2.6.6. Determination of Serum and Jejunal Mucosal Antioxidant Capacity

Jejunal mucosa samples were accurately weighed and homogenized in ice-cold physiological saline at a ratio of 1:10 (*w*/*v*) using a high-speed homogenizer at 2500 r/min for 10 min. The homogenate was centrifuged at 3500 r/min for 10 min at 4 °C, and the supernatant was collected for analysis.

The activities of serum superoxide dismutase (SOD), GSH-Px, and CAT, as well as the contents of glutathione (GSH) and malondialdehyde (MDA) in serum and jejunal mucosal homogenates, were measured using commercial kits (Nanjing Jiancheng Bioengineering Institute). GR activity was determined only in jejunal mucosal homogenates. All procedures were strictly followed according to the manufacturer’s instructions.

#### 2.6.7. Assessment of Jejunum Oxidative Damage Level

The content of 8-OHdG in jejunal tissue was determined using a commercial ELISA kit (Nanjing Jiancheng Bioengineering Institute) according to the manufacturer’s protocol. PC content was measured following the method described by Wei et al. [24].

### 2.7. Statistical Analysis

All experimental data were compiled using Excel 2021 (Microsoft Corp., Redmond, WA, USA). Normality was assessed using the Shapiro–Wilk test, and homogeneity of variance was evaluated using Levene’s test. As the data satisfied the assumptions of normality and homogeneity of variance, one-way analysis of variance (ANOVA) was performed, followed by Duncan’s post hoc test using SPSS version 27.0 (IBM Corp., Armonk, NY, USA). Data are presented as means with standard error of the mean (SEM). Statistical significance was set at *p* < 0.05, while *p* < 0.01 was considered highly significant.

## 3. Results

### 3.1. Growth Performance

As shown in Table 2, lambs in the IUGR group had significantly lower initial body weight, final body weight, and average daily gain than those in the CON group (*p* < 0.01). Compared with the IUGR group, lambs supplemented with DMG-Na exhibited highly significantly increased final body weight and significantly increased average daily gain (*p* < 0.01). No significant difference was observed in initial body weight between the IUGR and IUGR + DMG-Na groups (*p* > 0.05).

### 3.2. Organ Indices

According to Table 3, compared with the CON group, lambs in the IUGR group had highly significantly lower liver and spleen weights (*p* < 0.01), and significantly lower heart, lung, and kidney weights, as well as spleen index (*p* < 0.05). Compared with the IUGR group, lambs in the IUGR + DMG-Na group exhibited highly significant increases in spleen weight and spleen index (*p* < 0.01), and a significant increase in liver weight (*p* < 0.05). No significant differences were observed in heart, liver, lung, or kidney indices among the groups (*p* > 0.05).

### 3.3. Small Intestinal Tissue Morphology

#### 3.3.1. Duodenal Tissue Morphology

**Figure 1:**
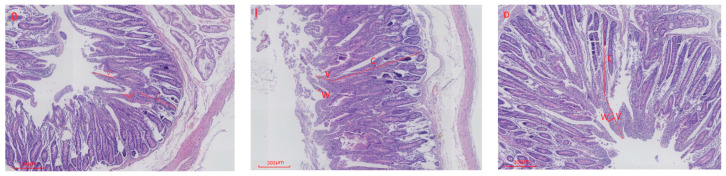
Comparison of duodenal morphology among CON, IUGR, and IUGR + DMG-Na lambs. O: CON group lambs; I: IUGR group lambs; D: IUGR + DMG-Na group lambs. V: villus height; W: villus width; C: crypt depth.

Electron microscopic observations of duodenal histomorphology in lambs from the CON, IUGR, and IUGR + DMG-Na groups are presented in Figure 1.

Data from Table 4 indicate that compared with the CON group, the IUGR group exhibited highly significant reductions in duodenal villus height and villus height-to-crypt depth ratio (*p* < 0.01), a highly significant increase in crypt depth (*p* < 0.01), and no significant difference in villus width (*p* > 0.05). Compared with the IUGR group, the IUGR + DMG-Na group exhibited highly significant increases in villus height, villus width, and villus height-to-crypt depth ratio (*p* < 0.01), and a highly significant decrease in crypt depth (*p* < 0.01).

#### 3.3.2. Jejunal Tissue Morphology

**Figure 2:**
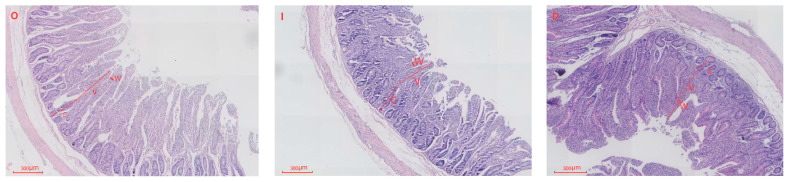
Comparison of jejunal morphology among CON, IUGR, and IUGR + DMG-Na lambs. O: CON group lambs; I: IUGR group lambs; D: IUGR + DMG-Na group lambs. V: villus height; W: villus width; C: crypt depth.

Electron microscopic images of jejunal tissue morphology in lambs from the CON, IUGR, and IUGR + DMG-Na groups are presented in Figure 2.

As shown in Table 5, compared with the CON group, lambs in the IUGR group exhibited highly significant reductions in jejunal villus height, villus width, and villus height-to-crypt depth ratio (*p* < 0.01), along with a highly significant increase in crypt depth (*p* < 0.01). Compared with the IUGR group, lambs in the IUGR + DMG-Na group showed highly significant increases in jejunal villus height, villus width, and villus height-to-crypt depth ratio (*p* < 0.01), and a highly significant decrease in crypt depth (*p* < 0.01).

#### 3.3.3. Ileal Tissue Morphology

**Figure 3:**
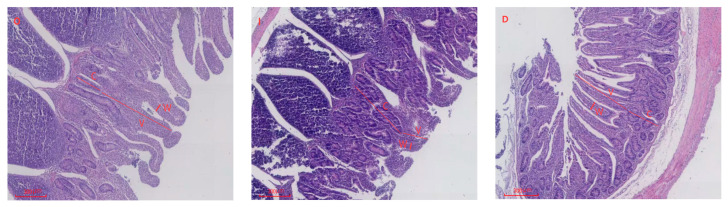
Comparison of ileal morphology among CON, IUGR, and IUGR + DMG-Na lambs. O: CON group lambs; I: IUGR group lambs; D: IUGR + DMG-Na group lambs. V: villus height; W: villus width; C: crypt depth.

Electron microscopic images of ileal tissue morphology in lambs from the CON, IUGR, and IUGR + DMG-Na groups are represented in Figure 3.

Consistent with the data in Table 6, compared with the CON group, lambs in the IUGR group exhibited highly significant reductions in villus height, villus width, and villus height-to-crypt depth ratio (*p* < 0.01), along with a highly significant increase in crypt depth (*p* < 0.01). Compared with the IUGR group, lambs in the IUGR + DMG-Na group showed highly significant increases in villus height, villus width (*p* < 0.01), and a significant increase in villus height-to-crypt depth ratio (*p* < 0.05).

### 3.4. Serum IgA, IgM, IgG, Jejunal sIgA, Endotoxin, and IGF-II

Table 7 shows that compared with the CON group, lambs in the IUGR group had highly significantly lower jejunal sIgA (*p* < 0.01), highly significantly higher endotoxins (*p* < 0.01) and significantly higher serum IgG (*p* < 0.05). Compared with the IUGR group, lambs supplemented with DMG-Na exhibited highly significant decreases in endotoxins (*p* < 0.01), significant decreases in serum IgG (*p* < 0.05), and significant increases in jejunal sIgA (*p* < 0.05).

### 3.5. Jejunum Digestive Enzymes

As shown in Table 8, compared with the CON group, lambs in the IUGR group had significantly lower activities of amylase, lipase, and maltase (*p* < 0.05). Compared with the IUGR group, lambs supplemented with DMG-Na exhibited significantly increased amylase, lipase, and maltase activities (*p* < 0.05), while sucrase, trypsin, and lactase activities were numerically higher but did not differ significantly (*p* > 0.05).

### 3.6. Antioxidant

#### 3.6.1. Antioxidant Capacity in Serum

According to Table 9, compared with the CON group, lambs in the IUGR group had significantly lower serum SOD, GSH, and GSH-Px activities (*p* < 0.05). Compared with the IUGR group, lambs supplemented with DMG-Na exhibited significantly increased serum SOD, GSH, and GSH-Px activities (*p* < 0.05).

#### 3.6.2. Antioxidant Capacity in Jejunum

Compared with the CON group, lambs in the IUGR group had significantly lower jejunal GSH, GR, and CAT activities (*p* < 0.05) and highly significantly higher jejunal MDA content (*p* < 0.01). Compared with the IUGR group, lambs supplemented with DMG-Na exhibited significantly increased jejunal GSH, and CAT activities (*p* < 0.05) and a highly significant decrease in jejunal MDA content (*p* < 0.01), while jejunal SOD and GSH-Px activity was numerically higher but did not differ significantly (*p* > 0.05) (Table 10).

#### 3.6.3. Oxidative Damage in the Jejunum

According to Table 11, compared with the CON group, lambs in the IUGR group exhibited highly significant increases in both 8-OHdG and PC content in the jejunum (*p* < 0.01). Compared with the IUGR group, lambs supplemented with DMG-Na showed highly significant reductions in both 8-OHdG and PC content in the jejunum (*p* < 0.01).

## 4. Discussion

### 4.1. Growth Performance

IUGR is typically associated with fetal malnutrition and incomplete development in utero, resulting in lower birth weight and impaired postnatal growth in lambs. The findings indicate that IUGR leads to reduced birth weight in lambs [25]. The present study demonstrated that lambs in the IUGR group had significantly lower initial body weight, final body weight, and average daily gain compared with the CON group. The present study confirmed that IUGR lambs have impaired growth performance compared to their normal counterpart [26]. Reduced initial weight reflects fetal growth restriction in utero, while the significant decline in final weight and average daily gain indicates markedly slower postnatal growth rates in IUGR lambs relative to the CON group. Compared with the IUGR group, lambs in the IUGR + DMG-Na group exhibited significantly higher final body weights and average daily weight gains, indicating that DMG-Na partially mitigated growth restriction caused by IUGR. Dietary supplementation with 1500 mg/kg DMG-Na significantly increased average daily weight gain in broiler chicken [27]. Studies have also demonstrated that dietary supplementation with DMG-Na significantly enhanced growth performance in IUGR piglets [28]. These findings suggest DMG-Na emulsifies nutrients in the small intestine to facilitate their digestion and absorption, thereby promoting growth in IUGR lambs [29].

### 4.2. Organ Index

The normal development of visceral organs is essential for physiological function, with organ mass serving as a crucial biological indicator. Organ indices reflect physiological status to a certain extent [30]. This study revealed highly significant reductions in liver and spleen weights in IUGR lambs, alongside significant decreases in heart, lung, and kidney weights, as well as spleen index. IUGR restricts liver development in suckling piglets. Under IUGR conditions, normal organ development is constrained, leading to hypoplasia across multiple organs [30]. Dietary supplementation with DMG-Na increases liver and spleen indices in IUGR piglets. Following feeding with DMG-Na-supplemented diets, IUGR lambs exhibited a highly significant increase in spleen weight and spleen index, alongside a significant rise in liver weight. These findings broadly align with prior studies, suggesting DMG-Na intervention may mitigate organ developmental impairment caused by IUGR. As the largest peripheral immune organ in animals and a vital component of the immune system, the spleen’s developmental status directly influences the proper functioning of humoral and cellular immunity, including lymphopoiesis, antigen presentation, and antibody production [31]. The increased spleen weight in IUGR + DMG-Na lambs reflects enhanced cellular growth, development, and proliferation, reflecting immune enhancement. This suggests DMG-Na may help alleviate the innate immune developmental deficits in IUGR individuals. The elevated spleen index may further indicate recovery of spleen volume and function [22].

### 4.3. Intestinal Tissue Morphology

The functional performance of the small intestine is intrinsically linked to its physiological structure; consequently, the tissue architecture of the intestinal mucosa is pivotal to an animal’s health status [32]. The villous architecture of the small intestine provides the morphological foundation for performing the core physiological function of nutrient digestion and absorption [33]. Increased villus height directly signifies an expansion of the mucosal surface area available for absorption, marking enhanced intestinal absorptive capacity [34,35]. Conversely, villus atrophy and fusion are typically closely associated with malabsorption [36]. The rich capillary network and lymphoid tissue within small intestinal villi provide the structural foundation for nutrient absorption and immune defense [37,38]. Consequently, increased villus height correlates with enhanced digestive and absorptive capacity. Furthermore, the elongated villus architecture forms a physical barrier that aids in repelling harmful pathogens [39]. Crypts serve as the proliferative centers for intestinal epithelial cells. Deepening crypts often reflect accelerated cellular proliferation to compensate for the loss of apical villus cells, or may indicate an imbalance in cellular renewal under pathological conditions [40].

Research indicates that IUGR decreases intestinal villus height in newborn piglets [41]. IUGR alters intestinal structure in pigs, reducing villus height, villus width, and villus-to-crypt ratio in the duodenum and jejunum [42]. Studies indicate that supplementing diets with 0.1% DMG-Na improves nutrient digestion and absorption in IUGR piglets [8]. In this study, IUGR lambs exhibited extremely significant reductions in duodenal villus height and villus-to-crypt ratio, alongside extremely significant increases in crypt depth. Similarly, jejunal and ileal villus height, villus width, and villus-to-crypt ratio were highly significantly diminished, while crypt depth was highly significantly elevated. These findings indicate that IUGR may lead to restricted small intestinal development. The severe reduction in villous surface area inevitably limits the efficiency of nutrient contact and uptake from colostrum and subsequent diets, contributing to the poor growth performance observed in IUGR lambs. The increased crypt depth in the IUGR group may relate to enhanced cellular proliferation; however fails to effectively restore villus function. This provides a structural explanation for the commonly observed postnatal phenomena of low nutrient utilization and growth developmental disorders. In this study, lambs in the IUGR + DMG-Na group exhibited highly significant increases in duodenal and jejunal villus height, villus width, alongside highly significant reductions in crypt depth. Similarly, ileal villus height, and villus width showed extremely significant increases. Furthermore, villus morphology appeared markedly more orderly and uniformly arranged, indicating enhanced digestive and absorptive capacity [43]. The synchronous and highly significant increases in villus height and width reflect an expansion of the absorptive surface area.

### 4.4. Immunity

IUGR restricts the development of the immune system in IUGR lambs [44]. This study showed that compared with the CON group, lambs in the IUGR group had extremely significantly lower jejunal sIgA levels, suggesting that IUGR impairs the immune function and intestinal mucosal immune barrier function of lambs. IgA (particularly intestinal sIgA) serves as a key effector molecule in mucosal immune defense, limiting pathogen adhesion and invasion through immune exclusion. Concurrently, serum IgG levels were significantly elevated in the IUGR group, which might be indicative of a passive enhancement of systemic immune responses against a backdrop of compromised mucosal barriers and heightened antigenic load. The significantly increased serum endotoxin level in the IUGR group, combined with the extremely significant decrease in jejunal sIgA, fully confirms that IUGR disrupts the mechanical and immune barrier functions of the lamb intestine, leading to substantial translocation of endotoxins produced by Gram-negative bacteria into the bloodstream [45]. The entry of endotoxins into the blood may activate the systemic humoral immune response as an immune adjuvant, which could be one of the reasons for the significantly higher serum IgG levels observed in the IUGR group. Compared with the IUGR group, dietary supplementation with DMG-Na significantly increased jejunal sIgA, indicating that DMG-Na partially restores intestinal mucosal immune function. This study demonstrates that dietary DMG-Na supplementation promotes the reconstruction of the intestinal mucosal immune barrier to some extent, thereby supporting intestinal health and the restoration of overall immune homeostasis.

### 4.5. Jejunal Digestive Enzyme

Amylase is a primary digestive enzyme responsible for hydrolyzing starch into small-molecule sugars directly absorbable by the intestine [46]. Lipase’s core function is to catalyze the hydrolysis of ester bonds in triglycerides, breaking down macromolecular fats into fatty acids and glycerol [47]. Maltase, an enzyme present on the small intestinal surface, further hydrolyzes maltose (produced from starch digestion) into glucose. Its active site is firmly anchored to the brush border membrane of intestinal epithelial cells [48]. Maltase activity levels directly reflect the integrity and functional state of mature absorptive cells at the apical end of intestinal villi [49]. Sucrase primarily acts to break down sucrose into the monosaccharides glucose and fructose. Sucrase frequently coexists with maltase in a complex form (sucrase-isomaltase complex) on the brush border membrane [50]. Consequently, its activity often exhibits high synergy with maltase activity, jointly reflecting the digestive integrity of the brush border. Trypsin belongs to the serine protease family [51]. This protein-digesting enzyme, secreted by the pancreas and entering the small intestine, breaks down proteins into smaller peptide chains, which are further hydrolyzed into amino acids, playing a central role in protein digestion. Lactase is the most crucial digestive enzyme for newborn and suckling lambs, breaking down lactose in milk replacer or colostrum into glucose and galactose [52]. Inadequate lactose digestion may cause diarrhea, bloating, and other digestive disturbances [53]. In the present study, the activities of amylase, lipase, and maltase were significantly lower in the jejunum of IUGR lambs compared with the control group, indicating that IUGR adversely affects digestive enzyme function as previously pointed out [41]. Digestive enzymes are key enzymes for intestinal digestion and nutrient absorption. Reduced activity of amylase, lipase, and maltase implies that IUGR lambs experience limitations in the digestion and absorption of carbohydrates, fats, and proteins. This may lead to malabsorption of nutrients, further impacting their growth and development. Compared with the IUGR group, the IUGR + DMG-Na group exhibited significantly elevated amylase, lipase, and maltase activities, providing more abundant material and energy substrates for growth. This improvement likely contributes to enhanced nutrient absorption and promotes growth.

### 4.6. Antioxidant Function

Redox reactions play a key role in amplifying physiological signals and pathophysiological processes, with endogenous antioxidant molecules participating in and regulating these responses. Free radicals and oxidants can be harmful to health and toxic to tissues and organs, contributing to cellular damage and organ dysfunction. Excessive production of these reactive species typically leads to structural and functional impairment of DNA, lipids, and proteins, thereby exerting long-term adverse effects on health [44]. Serum antioxidant markers reflect the systemic redox state, integrating the antioxidant capacity of key metabolic organs such as the liver, the extent of tissue oxidative stress, and the balance of antioxidants in circulation. Impaired antioxidant capacity is a key factor contributing to oxidative stress in IUGR lambs [54]. Oxidative damage elevates ROS levels within the body, diminishes antioxidant capacity, and disrupts mitochondrial structure [8]. SOD mitigates oxidative stress by catalyzing the dismutation of superoxide anions into hydrogen peroxide, which is subsequently neutralized by GSH-Px. GSH, a key intracellular non-enzymatic antioxidant, exerts protective effects by scavenging free radicals, reducing peroxides, and preserving protein sulfhydryl groups [55]. Elevated reactive oxygen species (ROS) load in IUGR lambs accelerates GSH depletion, while impaired GSH regeneration further reduces serum GSH levels. As one of the organs most exposed to the external environment, the intestine is particularly vulnerable to oxidative stress. Under IUGR conditions, the gut may be among the first targets of oxidative damage. In the present study, lambs in the IUGR group exhibited significantly reduced activities of GSH, GR, and CAT in the jejunum, along with markedly elevated MDA levels. Elevated MDA levels directly confirm substantial damage to the intestinal mucosal cell membrane structure and provide a molecular basis for the morphological alterations observed in IUGR lambs, including villus atrophy, crypt deepening, and increased intestinal permeability. Reduced CAT activity indicates impaired hydrogen peroxide scavenging, while decreased GSH and GR levels suggest insufficient glutathione cycling and antioxidant supply. Together, these changes result in localized ROS accumulation in the mucosa, confirming that the jejunum of IUGR lambs is under significant oxidative stress. Dietary supplementation with DMG-Na significantly increased jejunal GSH and CAT activities and decreased MDA levels in IUGR lambs. These findings suggest that DMG-Na mitigates oxidative damage by scavenging excess free radicals and enhancing immune defense mechanisms [15]. DMG has been shown to function as an antioxidant, enhancing the body’s antioxidant capacity [56]. DMG-Na acts as a direct glycine precursor and a key substrate for glutathione synthesis, supplying essential raw materials for de novo GSH synthesis and thereby improving redox status [56]. DMG-Na has been shown to improve offspring growth performance by mitigating oxidative damage induced by excessive ROS production [57]. DMG-Na has been reported to enhance oxygen utilization, thereby protecting against excessive free radical generation and improving immune status [58]. This study suggests that DMG-Na may enhance antioxidant capacity by scavenging excess ROS and thereby maintaining intracellular redox balance.

A dynamic equilibrium exists between ROS levels and the antioxidant system. However, this balance can be disrupted under conditions that induce oxidative stress. Excessive ROS production may lead to mitochondrial and DNA damage, ultimately impairing antioxidant capacity [59]. IUGR is closely associated with oxidative damage, mitochondrial dysfunction, and elevated ROS levels. 8-OHdG serves as a specific biomarker of DNA oxidative damage, formed when ROS attack the C-8 position of guanine bases [60]. Elevated 8-OHdG levels directly reflect increased oxidative damage to intracellular DNA. PC serves as a universal marker of protein oxidation, formed either through a direct ROS attack on amino acid side chains (particularly lysine, arginine, and proline) or via oxidative introduction of carbonyl groups. In the present study, both markers were significantly elevated in the jejunum of IUGR lambs, indicating the presence of oxidative stress. These findings are consistent with previous reports that IUGR induces oxidative damage in multiple tissues and organs, including the liver and intestine [22,61].

Compared with the IUGR group, lambs supplemented with DMG-Na exhibited significantly reduced levels of 8-OHdG and PC in the jejunum, approaching those observed in the CON group. This finding indicates that dietary DMG-Na supplementation effectively mitigates IUGR-induced intestinal oxidative damage. The reduction in these oxidative damage markers may be partially attributed to enhanced antioxidant enzyme activity. Additionally, the tertiary amine group in the DMG molecule possesses reductive properties, which may lower lipid peroxidation products and attenuate free radical attacks on biomolecules [57]. As a methyl donor, DMG participates in the synthesis of mitochondrial phospholipids, including cardiolipin, which is essential for maintaining the activity and structural integrity of electron transport chain complexes within the inner mitochondrial membrane [62]. Improved mitochondrial function reduces electron leakage, thereby decreasing ROS generation at its source. Additionally, as a methyl donor, DMG may influence DNA and protein methylation states by maintaining intracellular S-adenosylmethionine levels, thereby regulating multiple inflammatory signaling pathways, including NF-κB. Consequently, DMG may suppress NF-κB activation through epigenetic mechanisms, reducing inflammatory cytokine release and mitigating inflammation-associated oxidative damage [63].

### 4.7. Limitations and Future Perspectives

Variations in maternal milk production and suckling behavior among lambs may have contributed to individual differences in nutrient intake. Therefore, the direct relationship between individual feed intake and growth performance or intestinal development could not be analyzed. Future studies should incorporate precise measurements of individual milk and feed intake to further elucidate the mechanisms of DMG-Na.

## 5. Conclusions

In conclusion, IUGR compromised growth performance, immunity, intestinal tissue morphology, and antioxidant function in lambs. Supplementation with 0.1% DMG-Na effectively alleviated these deficits, improving growth and intestinal health. The beneficial effects of DMG-Na appear to involve two complementary mechanisms: strengthening intestinal antioxidant defense, and enhancing intestinal structure and digestive enzyme activity to facilitate nutrient absorption.

## Figures and Tables

**Table 1:** Composition and nutritional components of the experimental diets (DM basis).

Items	Lamb Starter Feed (%)
Corn	51.50
Soybean meal	28.00
Bran	18.00
Limestone	2.0
Premix ^(1)^	0.5
NaCl	1.0
Total	100
Crude protein (CP)	18.32
Crude fat (EE)	2.19
Crude fiber (CF)	7.32
Crude (ASH)	6.23
Ca	1.39
P	0.36

^(1)^ The premix provided the following per kg of diets: S, 200 mg. Fe, 25 mg, Zn, 40 mg, Cu, 8 mg, Mn, 40 mg, I, 0.3 mg. Se, 0.2 mg, Co, 0.1 mg, VA, 940 IU, VD, 111 IU, VE, 20 IU.

**Table 2:** Effects of DMG-Na on growth performance of IUGR lambs (*n* = 15).

Items	Groups			SEM	*p* Value
	CON	IUGR	IUGR + DMG-Na		
Initial body weight/kg	6.56 ^Aa^	5.16 ^Bb^	5.31 ^Bb^	0.13	<0.001
Final body weight/kg	19.41 ^Aa^	14.86 ^Cc^	17.18 ^Bb^	0.35	<0.001
Average daily gain (g/d)	262.24 ^Aa^	198.03 ^Bc^	242.11 ^Ab^	5.21	<0.001

Note: CON: Normal birth weight lambs supplemented with milk replacer. IUGR: Intrauterine growth restricted (IUGR) lambs supplemented with milk replacer. IUGR + DMG-Na: Intrauterine growth restricted (IUGR) lambs supplemented with milk replacer containing 0.1% dimethylglycine sodium salt (DMG-Na). In the same row, values with no letter or the same letter superscripts indicate no significant difference (*p* > 0.05); while different small letter superscripts indicate significant difference (*p* < 0.05), and different capital letter superscripts indicate significant difference (*p* < 0.01).

**Table 3:** Effects of DMG-Na on internal organ indices in IUGR lambs.

Items	Groups			SEM	*p* Value
	CON	IUGR	IUGR + DMG-Na		
Heart weight, g	91.60 ^a^	72.78 ^b^	84.80 ^ab^	2.99	0.026
Liver weight, g	437.08 ^Aa^	339.38 ^Bb^	404.85 ^ABa^	13.03	0.003
Spleen weight, g	33.67 ^Aa^	23.07 ^Bb^	32.95 ^Aa^	1.31	<0.001
Lungs weight, g	262.48 ^a^	215.50 ^b^	237.63 ^ab^	7.10	0.018
Kidney weight, g	74.40 ^a^	59.13 ^b^	70.20 ^ab^	1.97	0.012
Heart index, %	0.47	0.47	0.48	0.01	0.821
Liver index, %	2.21	2.18	2.31	0.36	0.320
Spleen index, %	0.17 ^ABa^	0.15 ^Bb^	0.19 ^Aa^	0.01	<0.001
Lungs index, %	1.34	1.39	1.36	0.03	0.720
Kidney index, %	0.38	0.38	0.40	0.01	0.264

Note: CON: Normal birth weight lambs supplemented with milk replacer. IUGR: Intrauterine growth restricted (IUGR) lambs supplemented with milk replacer. IUGR + DMG-Na: Intrauterine growth restricted (IUGR) lambs supplemented with milk replacer containing 0.1% dimethylglycine sodium salt (DMG-Na). In the same row, values with no letter or the same letter superscripts indicate no significant difference (*p* > 0.05); while different small letter superscripts indicate significant difference (*p* < 0.05), and different capital letter superscripts indicate significant difference (*p* < 0.01).

**Table 4:** Effects of DMG-Na on duodenal tissue morphology in IUGR lambs.

Items	Groups			SEM	*p* Value
	CON	IUGR	IUGR + DMG-Na		
Duodenal villus height, μm	366.39 ^Aa^	273.00 ^Bb^	372.99 ^Aa^	11.18	<0.001
Duodenal villus width, μm	43.88 ^Bb^	40.86 ^Bb^	62.29 ^Aa^	1.78	<0.001
Duodenal crypt depth, μm	273.63 ^Bc^	496.52 ^Aa^	356.50 ^Bb^	17.61	<0.001
Duodenal VH/CD	1.6 ^Aa^	0.63 ^Bc^	1.13 ^Bb^	0.08	<0.001

Note: CON: Normal birth weight lambs supplemented with milk replacer. IUGR: Intrauterine growth restricted (IUGR) lambs supplemented with milk replacer. IUGR + DMG-Na: Intrauterine growth restricted (IUGR) lambs supplemented with milk replacer containing 0.1% dimethylglycine sodium salt (DMG-Na). In the same row, values with no letter or the same letter superscripts indicate no significant difference (*p* > 0.05); while different small letter superscripts indicate significant difference (*p* < 0.05), and different capital letter superscripts indicate significant difference (*p* < 0.01).

**Table 5:** Effects of DMG-Na on jejunal tissue morphology in IUGR lambs.

Items	Groups			SEM	*p* Value
	CON	IUGR	IUGR + DMG-Na		
Jejunal villus height, μm	521.09 ^Aa^	264.88 ^Cc^	373.90 ^Bb^	15.38	<0.001
Jejunal villus width, μm	132.37 ^Aa^	63.03 ^Bc^	105.92 ^Ab^	5.99	<0.001
Jejunal crypt depth, μm	392.66 ^Bb^	590.72 ^Aa^	333.58 ^Bb^	18.28	<0.001
Jejunal VH/CD	1.48 ^Aa^	0.48 ^Bb^	1.29 ^Aa^	0.07	<0.001

Note: CON: Normal birth weight lambs supplemented with milk replacer. IUGR: Intrauterine growth restricted (IUGR) lambs supplemented with milk replacer. IUGR + DMG-Na: Intrauterine growth restricted (IUGR) lambs supplemented with milk replacer containing 0.1% dimethylglycine sodium salt (DMG-Na). In the same row, values with no letter or the same letter superscripts indicate no significant difference (*p* > 0.05); while different small letter superscripts indicate significant difference (*p* < 0.05), and different capital letter superscripts indicate significant difference (*p* < 0.01).

**Table 6:** Effects of DMG-Na on ileal tissue morphology in IUGR lambs.

Items	Groups			SEM	*p* Value
	CON	IUGR	IUGR + DMG-Na		
Ileal villus height, μm	458.61 ^Aa^	305.75 ^Bb^	441.25 ^Aa^	13.39	<0.001
Ileal villus width, μm	53.08 ^Aa^	40.17 ^Bb^	54.36 ^Aa^	1.54	<0.001
Ileal crypt depth, μm	258.44 ^Bb^	423.38 ^Aa^	417.29 ^Aa^	13.05	<0.001
Ileal VH/CD	1.98 ^Aa^	0.79 ^Bc^	1.12 ^Bb^	0.08	<0.001

Note: CON: Normal birth weight lambs supplemented with milk replacer. IUGR: Intrauterine growth restricted (IUGR) lambs supplemented with milk replacer. IUGR + DMG-Na: Intrauterine growth restricted (IUGR) lambs supplemented with milk replacer containing 0.1% dimethylglycine sodium salt (DMG-Na). In the same row, values with no letter or the same letter superscripts indicate no significant difference (*p* > 0.05); while different small letter superscripts indicate significant difference (*p* < 0.05), and different capital letter superscripts indicate significant difference (*p* < 0.01).

**Table 7:** Serum IgA, IgM, IgG, jejunal sIgA, endotoxin, and IGF-II.

Items	Groups			SEM	*p* Value
	CON	IUGR	IUGR + DMG-Na		
IgA, mg/mL	2.97	2.28	2.85	0.16	0.087
IgM, mg/mL	1.96	2.02	2.06	0.10	0.921
IgG, mg/mL	7.21 ^b^	9.86 ^a^	6.59 ^b^	0.47	0.003
sIgA, μg/mg protein	4.50 ^Aa^	3.18 ^Bc^	3.59 ^Bb^	0.20	<0.001
Endotoxin, EU/mL	0.90 ^Bb^	2.10 ^Aa^	0.96 ^Bb^	0.19	<0.001
IGF-II, ng/mL	16.87	16.05	16.58	0.44	0.771

Note: CON: Normal birth weight lambs supplemented with milk replacer. IUGR: Intrauterine growth restricted (IUGR) lambs supplemented with milk replacer. IUGR + DMG-Na: Intrauterine growth restricted (IUGR) lambs supplemented with milk replacer containing 0.1% dimethylglycine sodium salt (DMG-Na). In the same row, values with no letter or the same letter superscripts indicate no significant difference (*p* > 0.05); while different small letter superscripts indicate significant difference (*p* < 0.05), and different capital letter superscripts indicate significant difference (*p* < 0.01).

**Table 8:** Effects of DMG-Na on jejunal digestive enzyme activity in IUGR lambs.

Items	Groups			SEM	*p* Value
	CON	IUGR	IUGR + DMG-Na		
Amylase, U/mg protein	2.11 ^a^	1.10 ^b^	2.09 ^a^	0.18	0.026
Lipase, U/g protein	22.27 ^a^	10.06 ^b^	19.63 ^a^	1.63	0.020
Maltase, U/mg protein	2.24 ^a^	1.29 ^b^	2.02 ^a^	0.14	0.007
Sucrase, U/mg protein	3.64	2.13	3.33	0.28	0.052
Trypsin, U/mg protein	263.13	248.05	259.60	14.36	0.915
Lactase, U/mg protein	38.12	37.15	40.27	0.87	0.350

Note: CON: Normal birth weight lambs supplemented with milk replacer. IUGR: Intrauterine growth restricted (IUGR) lambs supplemented with milk replacer. IUGR + DMG-Na: Intrauterine growth restricted (IUGR) lambs supplemented with milk replacer containing 0.1% dimethylglycine sodium salt (DMG-Na). In the same row, values with no letter or the same letter superscripts indicate no significant difference (*p* > 0.05); while different small letter superscripts indicate significant difference (*p* < 0.05).

**Table 9:** Effects of DMG-Na on serum antioxidant capacity in IUGR lambs.

Items	Groups			SEM	*p* Value
	CON	IUGR	IUGR + DMG-Na		
SOD, U/mL	146.76 ^a^	123.91 ^b^	147.78 ^a^	3.88	0.011
GSH-Px, U/mL	246.08 ^a^	175.90 ^b^	244.25 ^a^	11.63	0.007
GSH, μmol/L	28.06 ^a^	17.72 ^b^	25.95 ^a^	1.45	0.002
CAT, U/mL	0.89	0.80	0.82	0.06	0.808
MDA, nmol/mL	2.18	2.79	2.13	0.13	0.061

Note: CON: Normal birth weight lambs supplemented with milk replacer. IUGR: Intrauterine growth restricted (IUGR) lambs supplemented with milk replacer. IUGR + DMG-Na: Intrauterine growth restricted (IUGR) lambs supplemented with milk replacer containing 0.1% dimethylglycine sodium salt (DMG-Na). In the same row, values with no letter or the same letter superscripts indicate no significant difference (*p* > 0.05); while different small letter superscripts indicate significant difference (*p* < 0.05).

**Table 10:** Effects of DMG-Na on jejunal antioxidant capacity in IUGR lambs.

Items	Groups			SEM	*p* Value
	CON	IUGR	IUGR + DMG-Na		
SOD, U/mg protein	167.92	142.93	168.87	5.24	0.062
GSH-Px, U/mg protein	24.15	21.25	23.32	1.41	0.719
GSH, μmol/g prot	20.48 ^a^	12.51 ^b^	20.16 ^a^	1.37	0.008
CAT, U/mg protein	2.11 ^a^	1.10 ^b^	2.09 ^a^	0.18	0.026
GR, U/g protein	1.02 ^a^	0.71 ^b^	0.87 ^ab^	0.05	0.023
MDA, nmol/mg protein	0.35 ^Bc^	0.81 ^Aa^	0.58 ^Bb^	0.06	<0.001

Note: CON: Normal birth weight lambs supplemented with milk replacer. IUGR: Intrauterine growth restricted (IUGR) lambs supplemented with milk replacer. IUGR + DMG-Na: Intrauterine growth restricted (IUGR) lambs supplemented with milk replacer containing 0.1% dimethylglycine sodium salt (DMG-Na). In the same row, values with no letter or the same letter superscripts indicate no significant difference (*p* > 0.05); while different small letter superscripts indicate significant difference (*p* < 0.05), and different capital letter superscripts indicate significant difference (*p* < 0.01).

**Table 11:** Effects of DMG-Na on jejunal oxidative damage in IUGR lambs.

Items	Groups			SEM	*p* Value
	CON	IUGR	IUGR + DMG-Na		
8-OHdG (ng/mL)	8.75 ^Bb^	12.44 ^Aa^	10.64 ^Bc^	0.48	<0.001
PC (nmol/mg prot)	2.37 ^Bb^	4.09 ^Aa^	2.97 ^Bb^	0.23	<0.001

Note: CON: Normal birth weight lambs supplemented with milk replacer. IUGR: Intrauterine growth restricted (IUGR) lambs supplemented with milk replacer. IUGR + DMG-Na: Intrauterine growth restricted (IUGR) lambs supplemented with milk replacer containing 0.1% dimethylglycine sodium salt (DMG-Na). In the same row, values with no letter or the same letter superscripts indicate no significant difference (*p* > 0.05); while different small letter superscripts indicate significant difference (*p* < 0.05), and different capital letter superscripts indicate significant difference (*p* < 0.01).

## Data Availability

The original contributions presented in the study are included in the article, further inquiries can be directed to the corresponding author.

## Abbreviations

The following abbreviations are used in this manuscript:

ADG	Average Daily Gain
CAT	Catalase
CBS	Cystathionine β-Synthase
CTH	Cystathionine γ-Lyase
DMG	Dimethylglycine
DMG-Na	Dimethylglycine Sodium Salt
GSH	Glutathione
GR	Glutathione Reductase
GSH-Px	Glutathione Peroxidase
H&E	Hematoxylin and Eosin
IUGR	Intrauterine Growth Restriction
IgA	Immunoglobulin A
IgM	Immunoglobulin M
IgG	Immunoglobulin G
MDA	Malondialdehyde
PC	Protein Carbonyl
ROS	Reactive Oxygen Species
sIgA	Secretory IgA
SOD	Auperoxide Dismutase
SAM	S-Adenosylmethionine
SAH	S-Adenosylhomocysteine
VH/CD	Villus Height-to-Crypt Depth Ratio
8-OHdG	8-Hydroxy-2′-Deoxyguanosine
